# Dissection guide for en bloc brachial and lumbosacral plexuses and central nervous system

**DOI:** 10.1007/s12565-026-00933-x

**Published:** 2026-05-09

**Authors:** Gail Elliott, Rebecca K. Lauber, Grace Pinhal-Enfield

**Affiliations:** 1 School of Graduate Studies, Rutgers University, Piscataway, NJ USA; 2 Department of Neuroscience and Cell Biology, Office of Education, Robert Wood Johnson Medical School, Rutgers University, Piscataway, NJ USA; 3 Department of Perioperative Services, The Mount Sinai Hospital, New York, NY USA; 4 Department of Neuroscience and Cell Biology, Anatomical Association, Robert Wood Johnson Medical School, Rutgers University, Piscataway, NJ USA

**Keywords:** En bloc dissection, Central nervous system, Brachial plexus, Lumbosacral plexus, Neuroanatomy education, Curriculum integration

## Abstract

Traditionally anatomy curricula often separate dissections of the central nervous system (CNS) and peripheral nerve plexuses, limiting opportunities for students to appreciate the nervous system as an integrated whole. Viewing the CNS and somatic components together offers the potential to enhance conceptual understanding, spatial reasoning, and clinical correlation. This guide details a step-by-step approach for the en bloc removal of the entire CNS with its associated brachial (C5-T1) and lumbosacral (L1-S4) plexuses. The dissection utilizes standard laboratory tools and follows a logical progression through the limbs, vertebral column, pelvis, and skull. Emphasis is placed on preserving the continuity of neural structures and minimizing damage during bone removal and blunt dissection. The resulting specimen demonstrates the full anatomical continuity from the brain to the terminal branches of the major limb nerves, allowing direct visualization of central-peripheral relationships. This preparation reveals key neuroanatomical pathways within a single integrated structure, potentially providing important teaching and demonstration value for gross anatomy and neuroscience courses. Although this dissection offers substantial pedagogical opportunities, it is not feasible for routine student performance due to its technical complexity and approximately 50-hour time requirement. Prolonged manual dissection and use of bone cutters also pose ergonomic challenges. However, when developed by trained faculty, this specimen may act as a high-impact educational resource that supports advanced neuroanatomical understanding and curriculum integration across medical and graduate anatomy programs.

## Introduction

Traditionally, gross anatomy students view the central nervous system (CNS), brachial plexus, and lumbosacral plexus in individual isolated dissections at various points within their curriculum (Detton 2024; Vugampore et al. 2025; Flack and Nicholson 2018). However, from an educational perspective, viewing these structures as one continuous single entity may offer substantial pedagogical value. This integrated approach would enable learners to conceptualize the nervous system as a unified network, extending from the central region to the peripheral targets, reinforcing the continuity between these components. By tracing pathways from the spinal cord through the plexuses to the limbs, students may also strengthen their three dimensional understanding of neuroanatomy and gain insight into the spatial organization of nerves in the human body. This visualization may also enhance deeper cognitive processes by promoting student learning to move beyond memorization towards constructing models of functional connectivity. Educationally, this integration may also contribute to both horizontal and vertical curricular alignment by linking foundational anatomy with clinical neuroscience, radiology, and surgery. Students may also learn to more readily predict the consequences of lesions at specific levels and potentially develop a mechanistic understanding of motor and sensory deficits. Ultimately, studying the CNS and major plexuses as a single structural unit potentially fosters both conceptual coherence and clinical reasoning, developing student’s appreciation of the nervous system’s complexity and design.

Historically, the development of complex anatomical dissections, including en bloc removal of the nervous system, has elicited considerable professional interest, yet such preparations remain uncommon within contemporary teaching institutions. In addition to their technical demands, these specimens have at times been subject to ethical scrutiny, particularly with respect to documentation of informed consent. A frequently cited example is the Harriet Cole nervous system preparation, currently stewarded by the Legacy Center Archives and Special Collections at Drexel University (Drexel University College of Medicine Legacy Center Archives and Special Collections 2026; Drexel University College of Medicine 2026). The specimen was prepared in 1888 by Dr. Rufus B. Weaver and has been the focus of historical debate concerning the circumstances of consent, given the era in which the dissection occurred and the absence of verifiable primary documentation (Drexel University College of Medicine 2026). Despite these controversies, the specimen has provided generations of medical learners with a unique opportunity to study the continuity of the human nervous system in situ and remains one of the few surviving preparations of its kind. This rarity is likely attributable to the substantial time investment required to produce such specimens, a misplaced perceived limited pedagogical value, and the technical value, and the technical difficulty of the procedure. Consequently, these specimens have become increasingly scarce, coinciding with a broader reduction in hands-on neuroanatomy dissections and laboratory hours, despite growing evidence that such experiences provide sufficient educational benefit (Drake et al. 2009; Chang and Molnár 2015; Arnts et al. 2014; Rae et al. 2016). Recent publications detailing the en bloc removal of the CNS (Hlavac et al. 2018, Hoffman et al. 2025) have underscored both the need for and the value of accessible instructional resources for generating these advanced neuroanatomical preparations. Hlavac and colleagues (Hlavac et al. 2018) piloted the educational use of an en bloc CNS specimen with graduate and medical student cohorts, reporting overwhelmingly positive feedback: 98% of participants found the specimen extremely useful and recommended its continued use, while medical students additionally noted its relevance for pre-clinical review. Building upon this work, Vugampore et al. (2025) produced a 19-minute instructional dissection video that guided students through the en bloc removal process. Students reported that performing the dissection significantly enhanced their understanding of neuroanatomy and affirmed the pedagogical value of such guides for developing proficiency with complex anatomical specimens in advanced dissection courses.

## Purpose

Recent studies on en bloc dissection of the central nervous system (CNS) (Hlavac et al. 2018; Hoffman et al. 2025) highlight both the pedagogical and integrative value of such specimens within gross anatomy, neuroscience, and neurology curricula. These findings underscore the importance of developing complex anatomical preparations that enhance cross-disciplinary learning and deepen students’ spatial understanding of the nervous system. Building on reported student feedback and appreciation for these specimens, future dissections could extend the en bloc CNS preparation to include additional neuroanatomical structures, specifically, the brachial and lumbosacral plexuses with their major terminal branches in the upper and lower limbs, respectively, to further promote integrated learning beyond the CNS. Accordingly, the objectives of this manuscript are two-fold: (1) to provide a detailed, step-by-step guide for the removal of the complete en bloc CNS with its associated brachial and lumbar plexuses, and (2) to make this complex dissection more attainable for anatomists seeking to preserve and advance the tradition of comprehensive neuroanatomical specimen preparation. Such dissections hold significant pedagogical potential across both anatomy and neuroanatomy curricula.

## Materials and methods

### Ethical approval and donor body preparation

The anatomical specimen described here was obtained from a human donor previously preserved for educational purposes. Ethical approval and institutional guidelines for the use of donated human material were followed from the in-house Anatomical Association. The specimen used in this study was obtained from an 84-year-old male donor whose cause of death was leukemia. The individual had voluntarily donated his body for educational and research purposes to the Anatomical Association at Robert Wood Johnson Medical School (New Jersey), in accordance with established ethical guidelines for reporting on human donors in academic manuscripts (Winkelmann et al. 2016). All identifying information has been removed to protect donor confidentiality. The individual provided informed consent for their body to be used for educational purposes, consistent with the intent of this work. The objective of this dissection guide is not to sensationalize the specimen or the individual, but rather to provide a methodological framework that may guide other institutions in undertaking similar dissections for internal educational use.

The donor specimen had been previously utilized for student-led anatomical dissection during a period of reduced donor availability that continues at the facility following the COVID-19 pandemic. As a result, certain thoracic structures, including portions of the intercostal nerves, had been partially disrupted prior to the initiation of this dissection and were therefore not included in the final en bloc preparation.

### Preservation method

Human donors were embalmed onsite using an in-house preservation solution consisting of phenol (15%), isopropyl alcohol (42.5%), and ethylene glycol (42.5%), supplemented with borax, potassium nitrate, and water. Injections of 300 ml of solution, through the inner canthus of the eye into the brain are carried out before they are left to settle for ~ 6 months. After this, they are introduced to the dissection lab. Finally, nervous tissue (brains, spinal cords, etc.) that are removed from the donor bodies are stored in a 25% ethylene glycol solution. This ethylene glycol solution is the in-house standard neural tissue preservative to minimize desiccation and inhibit microbial or fungal proliferation. When stored in this ethylene glycol–based solution, which serves as the in-house preservation medium for neuroanatomical materials, specimens are anticipated to remain viable for approximately three to five years. Longevity is influenced by the extent and nature of handling; minimizing direct student manipulation and ensuring appropriate faculty supervision during demonstrations can substantially extend the functional lifespan of the specimen. Ultimately, durability is contingent upon adherence to established handling protocols and the level of oversight provided during student interaction.

### Instruments

The instruments used for this dissection are commonly available in most anatomical laboratories and included a size 4 scalpel, scissors (Stevens Tetonomy Straight Scissors), forceps, bone cutters (Horsley Bone Cutters), and a bone saw (Mopec BD040 Autopsy saw). Nerve isolation and visualization were primarily achieved through blunt dissection using forceps and scissors. The bone saw was employed sparingly, for procedures, such as craniotomy or bone scoring, while the majority of bone cutting was performed manually using bone cutters to reduce the risk of damage to neuroanatomy tissues.

### Process

The process being described below to remove this specimen took one advanced dissector with approximately 16 years of dissection experience ~ 50 h over a two-week period in 3-hour blocks. This process of removal is based on six central nervous systems, and three brachial plexus-spinal cord removals, prior to attempting this dissection.

## Description

For the purposes of describing the next steps of the dissection, they will be separated into the limb dissections, skull and vertebral column, abdomen, and pelvis, providing a sequential and logical approach to the removal of this specimen.

### The limb dissections

Dissection of the limbs required deliberate planning to determine the extent to which the nerve plexuses would be incorporated into the final specimen. In this initial preparation, the principal terminal branches of the brachial and lumbosacral plexuses supplying the upper and lower limbs were identified and preserved. Each nerve was traced from its most proximal accessible point distally toward the digits, carefully isolating it from surrounding fascia and selectively transecting muscular branches to maintain continuity of the main trunks. These procedures were performed bilaterally on all limbs, as described in detail below.

The purpose of these initial steps was to identify and expose the nerves in preparation for their detachment during the final stages of dissection. Complete detachment at this stage was avoided to prevent potential damage during repositioning of the body between prone and supine orientations.

### The upper limb

The human donor was placed in supine position for these initial steps:

1. Bilaterally, the skin was removed from the anterior and lateral aspects of the neck, pectoral region, and entire limb, extending distally to the tips of the digits (Detton 2024).
2. In the neck region, the deep fascia and platysma muscle were incised, and the sternocleidomastoid muscle was detached from its clavicular and sternal attachments (Detton 2024).
3. At the root of the neck, the anterior scalene muscle was identified, with the phrenic nerve coursing along its anterior surface and the roots of the brachial plexus emerging through the interscalene triangle.
4. The phrenic nerve was carefully separated from the surface of the anterior scalene muscle to prevent injury during subsequent dissection. It was then traced proximally to its cervical roots before the anterior scalene muscle was incised to expose the roots of the brachial plexus (C5-T1) emerging from their respective intervertebral foramina.
5. From the roots of the brachial plexus, the nerve trunks were traced laterally through the neck toward the axilla. The clavicle was reflected, and the subclavian artery and vein were removed to allow clear visualization and continuation of the plexus into the axillary region.
6. In the axilla, the axillary fascia was removed and the divisions and cords of the brachial plexus were first identified and then followed distally to their respective terminal branches. From the lateral and medial cords, the formation of the median nerve was traced, while the ulnar nerve and the medial cutaneous brachial and antebrachial nerves were identified as branches of the medial cord and followed distally along their courses. The median nerve was traced along its course to the cubital fossa, while the ulnar nerve was traced behind the medial epicondyle of the humerus. The cutaneous branches were followed and detached at their most distal locations to prevent their damage through being continually anchored to cutaneous fascia and skin.
7. The posterior cord was subsequently identified, and the axillary artery and vein removed to enhance visualization. The posterior cord was traced laterally to the lateral border of the axilla, where it divided into its terminal branches: the axillary and radial nerves. The axillary nerve was followed along its course into the posterior shoulder as far as possible, while the radial nerve was traced to the point where it passed posterior to the humerus. The arm was then elevated to access the posterior compartment, and the radial nerve was further exposed within the radial groove of the humerus by transecting the lateral head of the triceps brachii muscle.
8. The ulnar nerve was then traced into the anterior compartment of the forearm by incising the common flexor attachments at the medial epicondyle. It was subsequently followed distally along its course between the flexor carpi ulnaris and flexor digitorum profundus muscles toward the hand (Detton 2024).
9. The median nerve was traced distally along its course from the cubital fossa by transecting the pronator teres muscle. Through the forearm, the median nerve was traced along its course between the flexor digitorum superficialis and flexor digitorum profundus muscles. The deep fascia of the forearm was reflected and the overlying flexor muscles were removed or reflected as necessary to enhance visualization. Just distal to the elbow joint, the anterior interosseous nerve branches from the median nerve and can be traced to be included in the specimen.
10. The radial nerve was traced from the posterior arm into the forearm as it coursed anterior to the lateral epicondyle, deep to the brachioradialis muscle. At this level, the nerve is divided into its superficial and deep branches, the latter giving rise to the posterior interosseous nerve. All branches were subsequently followed distally to ensure their preservation within the specimen, with overlying fascia removed and muscles incised as necessary to enhance visualization.
11. At the level of the wrist, the median nerve was traced through the carpal tunnel by incising the flexor retinaculum and subsequently followed into the palmar aspect of the hand.
12. To trace the ulnar nerve into the palmar aspect of the hand, the roof of Guyon’s canal, formed by the volar (palmar) carpal ligament and the palmaris brevis muscle, was incised to expose the nerve, which was then followed proximally into the forearm (Detton 2024).
13. The median and ulnar nerves were carefully traced through the palmar aspect of the hand. To facilitate this, the palmar aponeurosis was meticulously removed, guided by prior identification of the underlying nerve courses to avoid injury to the delicate branches situated immediately beneath it.
14. The common palmar digital branches of both nerves were carefully traced to their proper palmar digital branches at the bases of the digits. These branches were then followed distally along the medial and lateral aspects of each digit to their terminal points (Detton 2024).

### The lower limb

15. The skin was removed from the medial, anterior, and lateral aspects of the entire lower limb. Dissection commenced proximally with identification of the inguinal ligament at the superior aspect of the anterior thigh (Detton 2024).
16. Blunt dissection was then performed distal to the inguinal ligament to define the borders of the femoral triangle and to locate the branches of the femoral nerve within the lateral third of it. Portions of the fascia lata and underlying musculature were reflected to expose all branches of the femoral nerve, including the saphenous nerve as it continued distally along the medial aspect of the leg.
17. In the medial aspect of the thigh, the adductor longus muscle was reflected to expose the underlying adductor brevis. The anterior branch of the obturator nerve was identified on the surface of the adductor brevis, carefully cleaned, and freed from the muscle. The adductor brevis was then transected to reveal the posterior branch of the obturator nerve. Both branches were subsequently traced proximally to the main obturator nerve at the level of the obturator foramen within the pelvis (Detton 2024).
18. In the lateral aspect of the leg, approximately two-thirds of the way up, the superficial branch of the fibular nerve was identified as it pierced the crural fascia. The nerve was then traced distally onto the dorsum of the foot, where its dorsal digital branches were carefully cleaned and preserved.
19. In the anterior compartment of the leg, the crural fascia was incised to expose the tendons of the flexor hallucis longus and flexor digitorum longus at the level of the ankle. These tendons were separated to identify the deep branch of the fibular nerve. The nerve was then traced distally onto the dorsum of the foot to its dorsal digital branches and followed proximally to its origin from the common fibular nerve just inferior to the head of the fibula. The anterior and lateral leg muscles were transected as necessary to improve visualization.
20. The body was positioned prone, and the skin was removed from the gluteal region and the posterior aspect of the entire lower limb. Fat and fascia were cleared to define the borders of the gluteus maximus muscle. The gluteus maximus was then detached from its attachment along the sacrum. During this reflection, the inferior gluteal nerve was transected at its insertion into the muscle to permit complete exposure of the underlying structures (Detton 2024).
21. The piriformis muscle was identified, and the superior gluteal nerve was traced superior to it, following its course through the plane between the gluteus medius and gluteus minimus muscles toward the tensor fascia lata muscle.
22. The sciatic nerve was traced from its emergence below the piriformis muscle through the posterior thigh to the popliteal fossa, where it bifurcated into the tibial and common fibular nerves. The posterior cutaneous nerve of the thigh was also identified and, if preservation was intended, traced from its origin below the piriformis along its course through the posterior thigh (Detton 2024).
23. The common fibular nerve was carefully traced distal to the head of the fibula as it curved anteriorly toward the anterior and lateral compartments of the leg. During this process, the attachments of the fascia lata and the biceps femoris muscle were transected to improve visualization and facilitate nerve exposure.
24. The tibial nerve was traced distally into the posterior compartment of the leg. To improve visualization, the medial and lateral heads of the gastrocnemius muscle, as well as the medial attachment of the soleus muscle, were transected. The nerve was followed through the plane between the superficial and deep muscle groups to the tarsal tunnel.
25. The flexor retinaculum was incised, and the tibial nerve was followed into the plantar aspect of the foot, deep to the plantar aponeurosis. The aponeurosis was then removed to permit tracing of the tibial nerve into its medial and lateral plantar branches, as well as their respective common and proper plantar digital branches.

### The back

The human donor was placed in a prone position for these steps:

26. The skin and fascia were removed from the back and posterior neck to expose the superficial layer of the back muscles and the musculature of the posterior shoulder (Detton 2024).
27. The trapezius muscles were reflected along their proximal attachments to the vertebral column and along their lateral attachments to the spine of the scapula, acromion process, and lateral clavicle.
28. The deltoid muscle was reflected from its attachment to the spine of the scapula, enabling identification and tracing of the axillary nerve. The nerve was detached from its insertion within the deltoid muscle and followed deeper to its entry point into the quadrangular space.
29. The rhomboids and serratus posterior superior muscles were reflected laterally. The latissimus dorsi was then released from its proximal attachments along the vertebral column and iliac crest. The underlying serratus posterior inferior was also detached from its vertebral attachments to expose the deeper erector spinae muscle group.
30. The erector spinae, semispinalis, and splenius muscles were reflected and carefully separated from the vertebral column in the back and neck to expose the spinous processes and laminae of the vertebrae. The overlying muscles and fascia of the sacral region were also removed to achieve complete visualization of the vertebral column. Additionally, the skin and fascia over the posterior aspect of the skull were removed at this stage (Detton 2024).

### The laminectomy

31. An autopsy saw was used to perform the laminectomy. In the initial stage, the spinous processes of vertebrae C2–L5 were removed, facilitating placement of the circular saw head against the laminae for more precise cutting. Optimal angulation of the saw head was achieved by aligning it with the transverse processes and leaning it at 45 degrees from the spinous process (Detton 2024). Following the initial cuts, a chisel was used to complete removal of the laminae, which were connected by intervening ligaments, including the ligamentum flavum. The C1 posterior arch was cut using the bone cutters (Detton 2024).
32. Access to the sacral canal was achieved by lightly scoring at the same width as the laminectomy and carefully removing the bone using a chisel and the bone cutters. The result was that the dural sac was visible to its most distal location and the filum terminale externum could be traced further distally to the coccyx.

The donor body was then placed in a supine position for the craniotomy and the abdominal dissection components.

### The craniotomy

33. The scalp and temporalis muscles were reflected and cleaned from the skull in preparation for the craniotomy. A scalpel was then used to delineate the circumferential incision line for the autopsy saw, positioned approximately at the level corresponding to the rim of a hat on the head (approximately, two centimeters superior to the supraorbital margin) (Detton 2024).
34. Circumferential cuts were made using the autopsy saw, taking care to detect the resistance change of the diploë to avoid penetrating too deeply and damaging the underlying brain tissue.
35. A T-tool was used to gently mobilize the calvaria from the skull, creating separation between the dura mater and the inner surface of the calvaria. Once loosened, the falx cerebri of the dura was incised anteriorly to allow access to the cranial cavity, and additional sagittal incisions were made with a scalpel to facilitate complete detachment of the dura from the calvaria, allowing the calvaria to be removed.

### The abdomen: gastrointestinal tract removal

36. The skin and fascia overlying the anterior abdominal wall were removed, and the anterior and lateral abdominal wall muscles were reflected to provide access to the abdominal cavity (Detton 2024).
37. Blunt dissection was performed to separate the fascia and peritoneum surrounding the distal sigmoid colon. Two ligatures were placed ~ 4 cm apart around the distal sigmoid colon, each secured tightly, and the colon was transected between them to minimize the risk of fecal contamination within the dissection field (Detton 2024).
38. The superior rectal artery, along with any remaining fascia and connective structures restricting elevation of the sigmoid colon, was then transected to allow mobilization of the segment from the pelvic cavity. The abdominal portion of the esophagus was identified and transected with scissors, followed sequentially by division of the celiac trunk, superior mesenteric artery, and inferior mesenteric artery.
39. The spleen and pancreas were gently freed using blunt dissection. The inferior vena cava was transected between the liver and diaphragm, and the ligaments and fascial connections anchoring the liver to the diaphragm were divided. Dissection then proceeded from proximal to distal, carefully severing any remaining fascial and mesenteric attachments to permit removal of the gastrointestinal tract from the abdominal cavity, while preserving the nerves of the posterior abdominal wall (Detton 2024).

### The lumbosacral plexus

40. The psoas major muscles were identified bilaterally, with the obturator nerve positioned medially and the femoral nerve laterally. The psoas major was carefully removed to allow tracing of both nerves along their proximal courses to their branching from the anterior rami exiting the intervertebral foramina. At this stage, the distal connections of the femoral and obturator nerves were also transected, and the nerves were gently pulled proximally, positioned into the iliac fossa and kept moist with wetted paper towels to preserve their integrity.

Passing the obturator nerve through the obturator foramen can be challenging due to the presence of surrounding fascia and the obturator membrane; therefore, careful dissection is required to avoid nerve damage.

41. The components of the lumbosacral plexus (L1–S4) were subsequently isolated, including the roots of L1-L4 and the lumbosacral trunk (L4, L5) as it coursed into the pelvis anterior to the sacroiliac joint. The roots of the sciatic nerve were traced posteriorly within the pelvis to the greater sciatic foramen, with surrounding fascia and vasculature carefully removed to prevent inadvertent damage to the nerve branches.

The donor body was then placed in the prone position to complete the craniotomy and address the next steps with the vertebral column and sacrum.

### The posterior skull

**Fig. 1:**
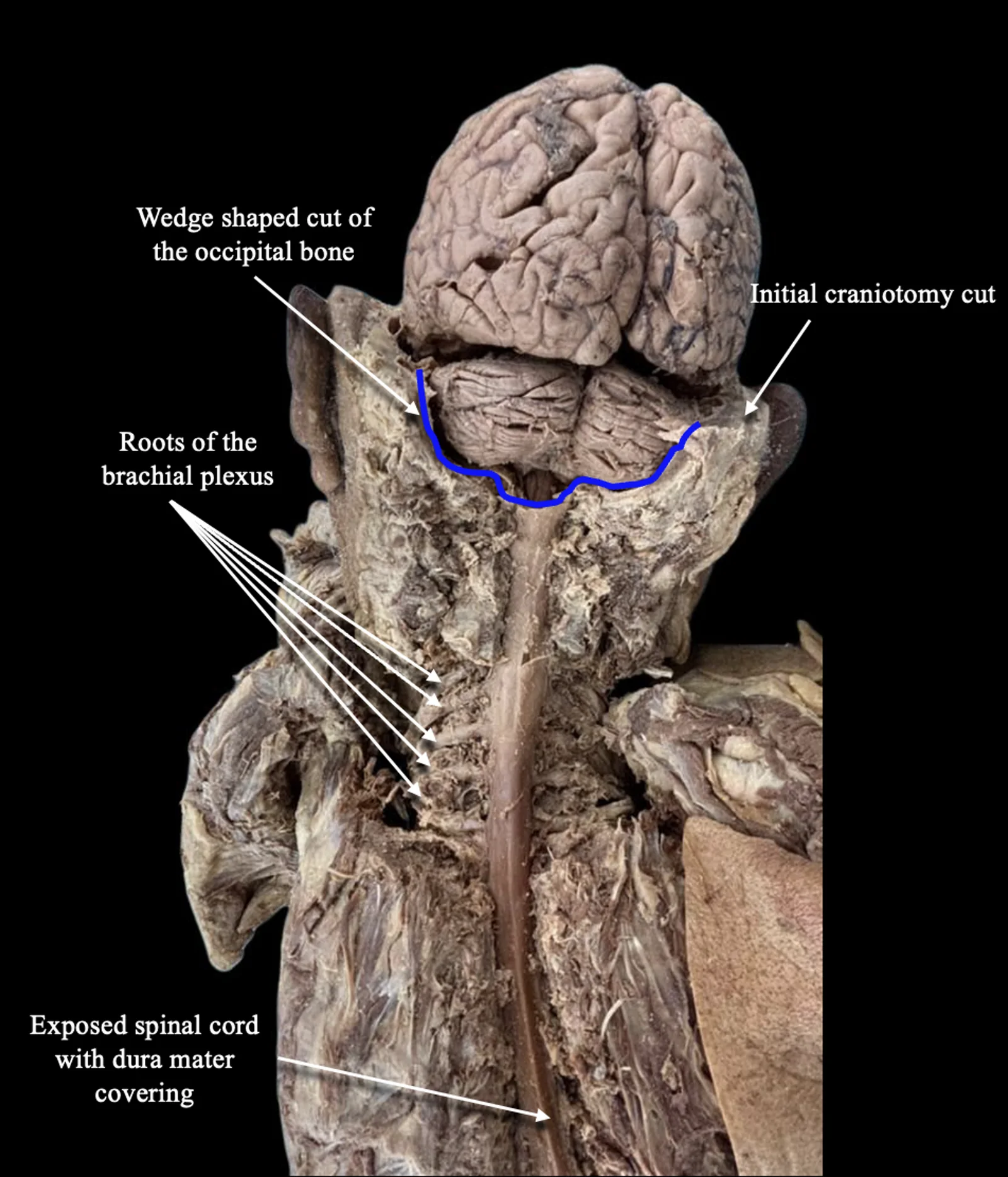
Posterior view of the human donor with the central nervous system exposed through the removal of the calvaria and the wedge of bone (denoted by blue line; mostly occipital). The roots of the brachial plexus (C5-T1) are also visible bilaterally

42. A wedge-shaped cut was made in the posterior aspect of the skull, extending from the margin of the initial calvarial section to the superior aspect of the vertebral column. This wedge was carefully removed, ensuring that the tentorium cerebelli and ligamentum craniale durae matris spinalis were incised and separated during the process (Fig. 1) (Ünal and Sezgin 2021).
43. The tentorium cerebelli was incised along the petrous portion of the temporal bone anteriorly toward the clivus of the occipital bone, and the posterior portion of the falx cerebri was transected at its junction with the tentorium cerebelli, which allowed for the complete removal of this cranial region of dura completely.
44. A visual inspection was conducted to confirm that no portions of the dura mater were restricting removal of the brain. The cranial nerves and vertebral arteries were then transected close to their points of attachment to the bone.

### The vertebral column

**Fig. 2:**
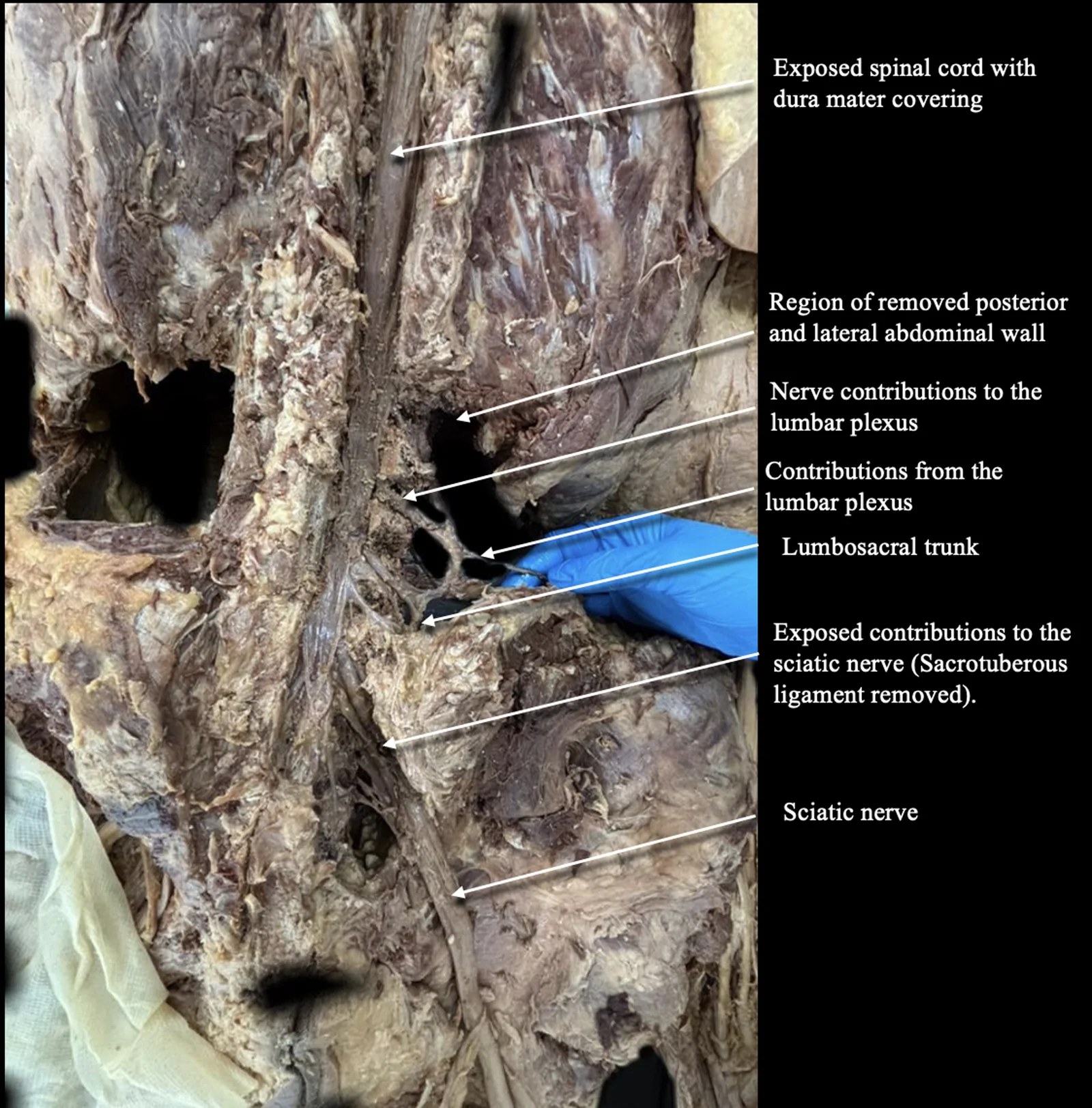
Posterior view of the donor, showing the exposed spinal cord in the dural covering, with the contributions to the lumbosacral plexus visible, and contributions to the sciatic nerve visible (L1-S4)

**Fig. 3:**
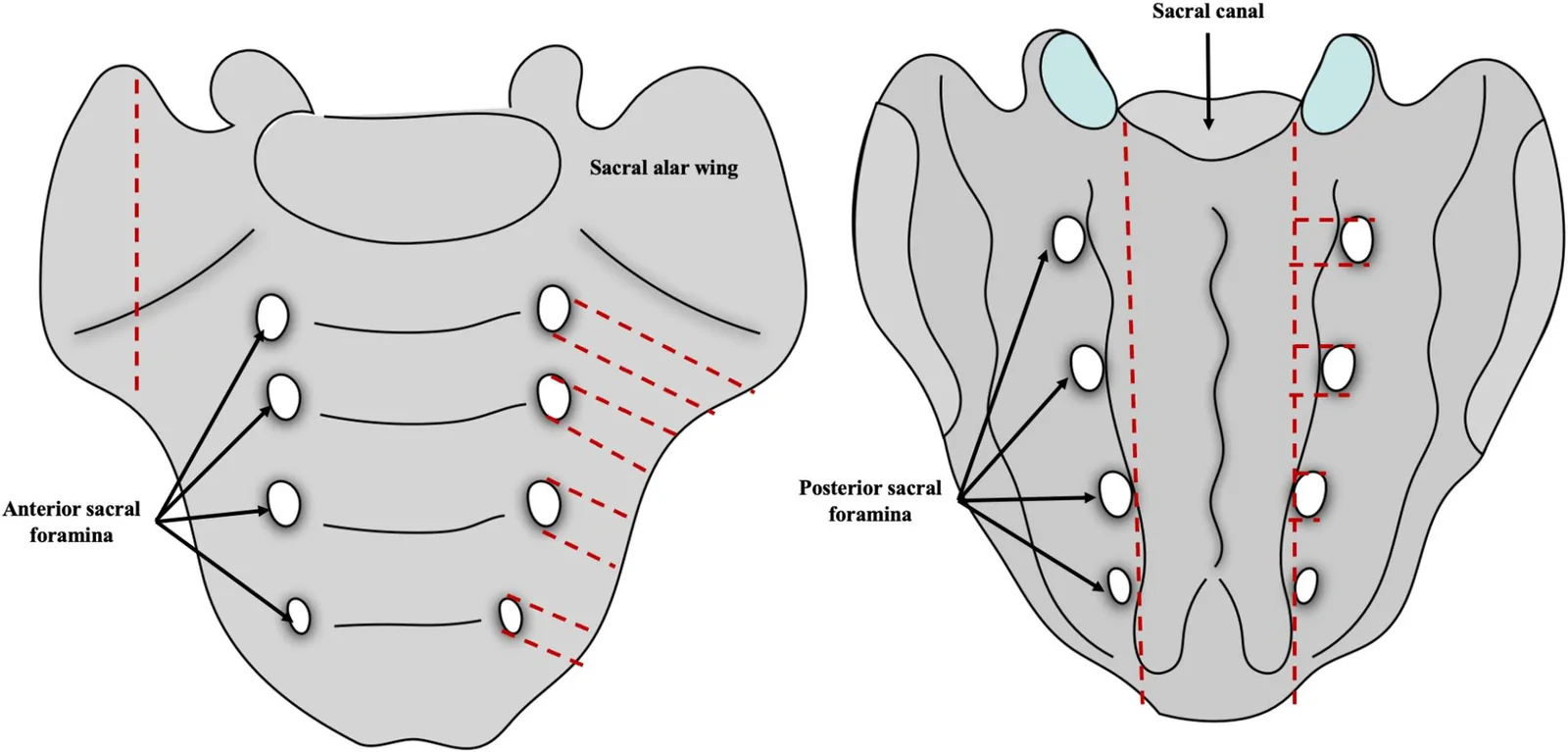
Osteotomies of the anterior (left) and posterior (right) sacrum. On the anterior aspect, complete transection of the sacral alar wings was performed to permit posterior mobilization of the neural elements and facilitate specimen removal. Additional cuts were made between the anterior sacral foramina and the lateral sacral margins to release the exiting nerves and allow their posterior displacement. On the posterior aspect, a laminectomy was undertaken, and the sacral canal was surgically connected to the sacral foramina. This approach enabled en bloc removal of the sacral contribution to the lumbosacral plexus in continuity with the remainder of the specimen. The red dotted lines show the locations where the cut marks would be made

45. The portions of the vertebral column between the laminectomy site and the intervertebral foramina were removed bilaterally along the length of the spine to expose the spinal nerves arising from the vertebral column. The bone was lightly scored with the autopsy saw and then removed using bone cutters to reveal the underlying neural structures. Care was taken to ensure clear visualization of the paired spinal nerves bilaterally, including the roots contributing to the brachial and lumbar plexuses, as well as any additional nerves designated for inclusion in the en bloc removal (see Fig. 2).
46. Bone cutters, along with a chisel and hammer applied with light taps, were used to trace the sacral nerve branches from the spinal cord through the anterior sacral foramina to their convergence forming the sciatic nerve. The sacrotuberous ligament was also removed during this process of tracing the contributions to the sciatic nerve (see Fig. 3).
47. The final stage is to remove a section of the pelvic bone between the pelvic cavity and the gluteal region, to allow for the lumbosacral trunk to be posterior to the pelvic bone with the sciatic nerve. Part of this step involved cutting through the abdominal wall laterally and posteriorly between rib 12 and the iliac crest to allow for visualization of the lumbar plexus and its branches from the posterior view, which proved to be extremely helpful.

At this stage, all paired spinal nerves were exposed and clearly visible from the posterior aspect of the specimen, with continuous visualization of the spinal cord within the dural sac and its contributions to the formation of the greater sciatic nerve. The lumbosacral trunk was also moved through the space created between the sacrum and iliac crest, to the gluteal region at the end of this stage.

### En bloc removal

The donor body was then placed in a supine position for the first part of the en bloc removal.

48. Beginning distally at the digits of the foot, the terminal branches of the superficial and deep fibular nerves were carefully released from their attachments to the surrounding musculature and skin using blunt dissection with scissors. Dissection proceeded proximally along the leg, ensuring that each nerve was preserved without traction or damage. At the level of the fibular neck, the common fibular nerve was identified, isolated, and freed from the surrounding muscles and fascia. The obturator and femoral nerves had been previously released during Step 40.
49. This procedure was repeated distally in the palmar aspect of the hand, where the branches of the median and ulnar nerves were traced proximally toward the brachial plexus and carefully freed along their courses. The medial brachial and medial antebrachial cutaneous nerves were also released at this stage.
50. The same process was performed for the radial nerve to ensure all three of its branches were fully isolated, along with the axillary nerve, before tracing the posterior cord proximally. Finally, the entire brachial plexus (C5-T1) was confirmed to be completely freed and followed to its roots at the intervertebral foramina.

The donor body was then placed in a prone position for the second part of the en bloc removal, with care to ensure that the loose terminal nerves were not trapped. Once this step was reached, a second table to put the specimen on, was moved close to the donor table.

**Fig. 4:**
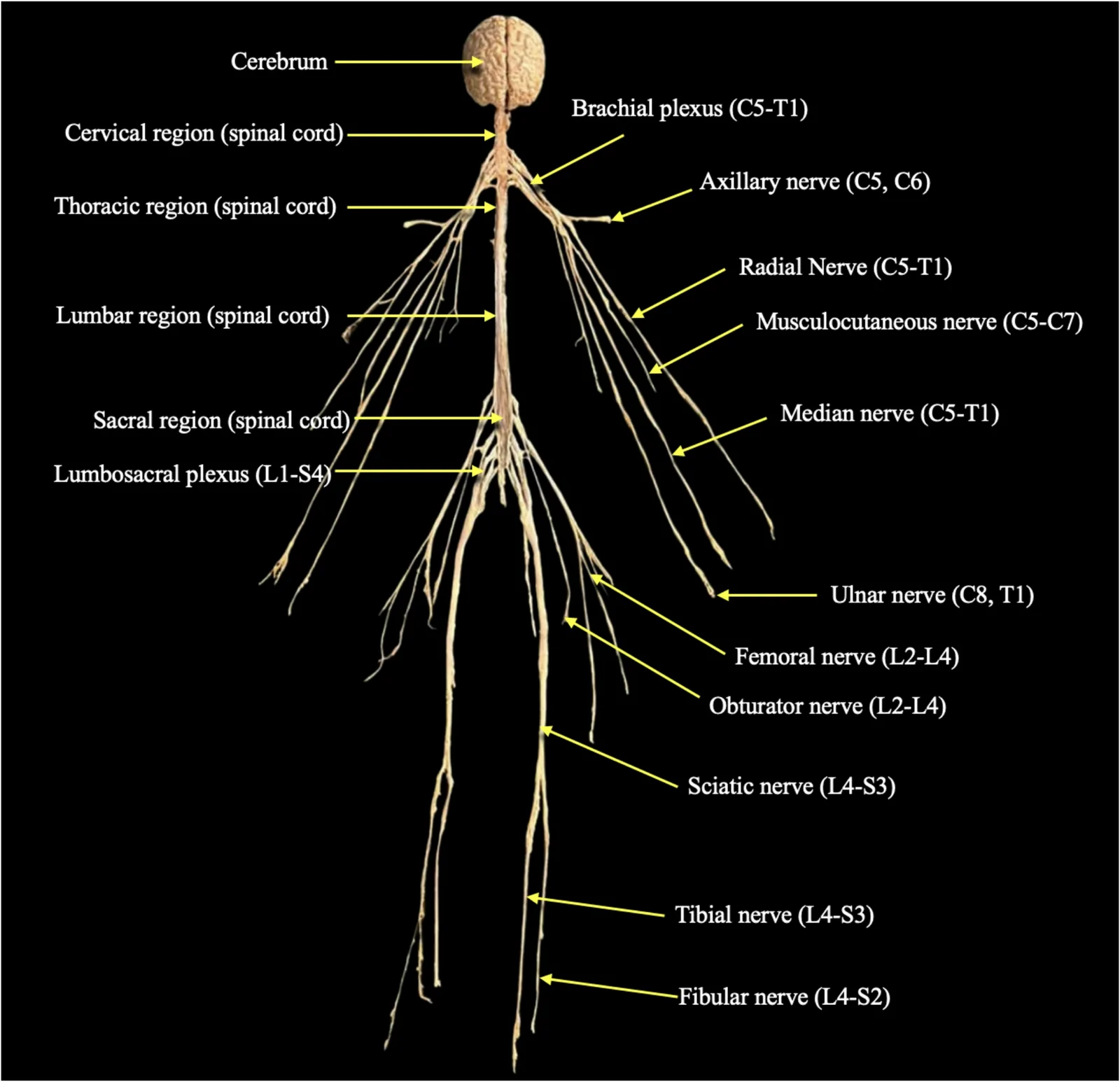
The anterior view of the final en bloc CNS and brachial and peripheral nervous plexuses after removal

51. Repeat this process for the tibial nerve by releasing the terminal digital branches of the plantar nerves and tracing them proximally through the tarsal tunnel to the tibial nerve, continuing upward to the popliteal fossa.
52. The dural sac was subsequently detached from the vertebral canal, beginning with transection of the filum terminale externum. The sac was then gently elevated while sequentially incising its connective attachments to the posterior longitudinal ligament, as well as associated structures within the region, including the anterior sacrodural ligament (Trolard’s ligament), Hoffman’s ligaments, and other fibrous slips (Ünal and Sezgin 2021; Barbaix et al. 1996). Given the high variability in the structural attachments of the dural sac, these steps were performed with particular caution.
53. Additional care was taken at the levels where spinal nerves exit to form the lumbosacral and brachial plexuses, as these structures were to be preserved within the specimen. Fibrous attachments known as the dural cuffs of Forestier, which anchor the spinal nerves to the bone of the intervertebral foramina, were carefully incised to ensure complete release of each individual spinal nerve (Barbaix et al. 1996).
54. After all ligamentous attachments had been incised, the spinal cord, along with the brachial and lumbosacral plexuses and their associated terminal nerves, were fully released. The final step involved the careful removal of the brain, after which the entire specimen was positioned on a preparation table (Fig. 4).

## Discussion

The en bloc dissection of the central nervous system and brachial and lumbosacral plexuses described in this guide offers a potential pedagogically-valuable opportunity to appreciate the full continuity of neural structures from the brain to the peripheral nerves of the upper and lower limbs. By integrating the cranial, spinal, and peripheral components into a single specimen, this approach enables visualization of the human nervous system, as a continuous, interconnected system, reinforcing both structural and functional relationships. This holistic view will likely support the development of students’ three-dimensional understanding of neuroanatomy and facilitation of clinical reasoning, by allowing learners to trace the origin and course of nerve fibers within a single specimen, something seldom achieved through standard dissection curricula. While previous studies have demonstrated the feasibility and educational value of en bloc CNS removal (Hlavac et al. 2018; Hoffman et al. 2025), the present guide expands upon that foundation by incorporating the brachial and lumbosacral specimens into a single specimen. The inclusion of these structures bridges the conceptual and physical gap between the central and peripheral nervous systems, allowing for simultaneous visualization of motor and sensory pathways from the spinal cord to their distal targets. Such an integrative approach can help students more effectively connect anatomical relationships with clinical correlates, such as patterns of neuropathy, or spinal cord injury.

### Limitations

Several important limitations should be considered when evaluating the educational utility of the en bloc CNS and peripheral limb nerve preparation. First, the process inherently involves the removal of surrounding musculature and associated tissues, effectively isolating the nervous system from its native anatomical context. While this isolation enhances visualization of neural continuity, it simultaneously eliminates key structural and functional relationships with the skeletal, cardiovascular, and muscular systems. As a result, learners may not be able to fully appreciate the integrated, systems-based interactions that are critical for comprehensive anatomical understanding.

Second, the technical complexity of producing this specimen substantially limits its feasibility within standard anatomy curricula. Preparation requires approximately 50 h of sustained, meticulous dissection, including extensive blunt dissection, bone removal, and dedicated tracing of neural structures across multiple anatomical regions. Such demands exceed what is practical for student-led activities in most medical, graduate, or undergraduate anatomy and physiology courses. Additionally, the repetitive manual use of scissors, forceps, and bone cutters introduces strain and potential occupational risk, underscoring the need for experienced anatomists to perform the procedure.

Third, although these preparations can function as long-term teaching resources, they require careful handling and appropriate storage conditions to preserve structural integrity. Fine neural structures are inherently delicate, and repeated handling across cohorts may increase the risk of damage without proper preservation protocols. Institutions adopting this approach must therefore ensure adequate laboratory infrastructure and specimen maintenance practices.

Finally, the specimen was derived from a donor body that had undergone prior student dissection, which resulted in partial disruption of some anatomical structures, including the intercostal nerves. Consequently, these nerves were not preserved in the final preparation.

### Educational value

From a pedagogical perspective, the integrated en bloc dissection offers an opportunity to integrate vertical and horizontal curriculum by linking foundational gross anatomy with neuroscience, neurology, and clinical medicine. The specimen’s continuity allows learners to correlate central lesions with peripheral manifestations, deepening comprehension of clinical syndromes, such as brachial plexus injuries. Additionally, the three-dimensional visualization of nerve pathways potentially supports a multimodal learning strategy that integrates spatial and visual aspects, key features shown to enhance long-term retention and conceptual mastery in anatomical sciences education (Drake et al. 2009; Chang and Molnár 2015; Arnts et al. 2014; Rae et al. 2016). Beyond the immediate anatomical content, exposure to such complex dissections potentially ingrains professional development by connecting students more deeply to human anatomy, donor altruism, and the traditions of anatomical inquiry.

This preparation also enables direct comparison between the brachial and lumbosacral plexuses, supporting learning of key organizational and functional differences between upper and lower limb innervation. For example, students may compare the structured root–trunk–division–cord organization of the brachial plexus with the more distributed branching pattern of the lumbosacral plexus, while simultaneously relating these to their spinal cord segmental origins. The specimen further allows learners to trace motor and sensory pathways from central origins to distal targets, reinforcing dermatomal and myotomal organization. From a clinical perspective, this integrated view may support comparison of injury patterns, such as upper limb plexus injuries (e.g., Erb’s or Klumpke’s palsies) and lower limb neuropathies (e.g., sciatic or femoral nerve injuries), facilitating prediction of functional deficits based on lesion location. This preparation may also support conceptual comparison between segmental and plexiform organization of peripheral nerves. While intercostal nerves were not preserved in the present specimen, their relatively linear course may be contrasted with the redistribution of fibers within the brachial and lumbosacral plexuses. This distinction may help learners understand dermatomal organization and its clinical relevance, particularly in differentiating radiculopathies from peripheral nerve injuries. In this context, students may appreciate that dermatomal patterns are more directly preserved in the thoracic region, whereas in the limbs, these relationships become more complex due to plexus formation and fiber redistribution.

Finally, to address the inherent limitation of isolating the nervous system from surrounding anatomical structures, this specimen may be most effective when integrated with complementary teaching modalities. For example, pairing the preparation with prosected specimens, radiological imaging (e.g., MRI or CT), or three-dimensional digital resources may help learners contextualize the spatial relationships between neural, musculoskeletal, and vascular systems. Although no notable anatomical abnormalities were identified in the present specimen, incorporation of clinically relevant variations or pathological findings, when available, may further enhance learner engagement and support clinical reasoning. In this way, the en bloc preparation is best positioned as part of a multimodal instructional approach rather than a standalone representation of anatomical relationships.

## Conclusion

In summary, the en bloc CNS and brachial and lumbosacral plexuses dissection presented here provides a unique and comprehensive visualization of neural connectivity in the human body. Although its technical difficulty and time demands preclude student-led completion, its creation by experienced anatomists yields a high-value educational specimen. By preserving and advancing the art of anatomical dissection through detailed documentation and modern dissemination, educators can continue to integrate tactile, conceptual, and clinical learning, ensuring that even the most complex neuroanatomical structures remain accessible to future generations of learners.

## Data Availability

No datasets were generated or analysed during the current study.
